# Prognostic and Theranostic Applications of Positron Emission Tomography for a Personalized Approach to Metastatic Castration-Resistant Prostate Cancer

**DOI:** 10.3390/ijms22063036

**Published:** 2021-03-16

**Authors:** Luca Filippi, Viviana Frantellizzi, Agostino Chiaravalloti, Mariano Pontico, Maria Silvia De Feo, Ferdinando Corica, Melissa Montebello, Orazio Schillaci, Giuseppe De Vincentis, Oreste Bagni

**Affiliations:** 1 Department of Nuclear Medicine, “Santa Maria Goretti” Hospital, Via Antonio Canova, 04100 Latina, Italy; o.bagni@ausl.latina.it; 2 Department of Radiological Sciences, Oncology and Anatomical Pathology, Sapienza University of Rome, Viale Regina Elena 324, 00100 Rome, Italy; viviana.frantellizzi@uniroma1.it (V.F.); mariano.pontico@uniroma1.it (M.P.); mariasilvia.defeo@uniroma1.it (M.S.D.F.); ferdinando.corica@uniroma1.it (F.C.); melissa.montebello@uniroma1.it (M.M.); giuseppe.devincentis@uniroma1.it (G.D.V.); 3 Department of Biomedicine and Prevention, University Tor Vergata, Viale Oxford 81, 00133 Rome, Italy; agostino.chiaravalloti@uniroma2.it (A.C.); orazio.schillaci@uniroma2.it (O.S.); 4 IRCCS Neuromed, 86077 Pozzilli, Italy

**Keywords:** castration-resistant prostate cancer, molecular imaging, positron emission computed tomography, theranostic nanomedicine

## Abstract

Metastatic castration-resistant prostate cancer (mCRPC) represents a condition of progressive disease in spite of androgen deprivation therapy (ADT), with a broad spectrum of manifestations ranging from no symptoms to severe debilitation due to bone or visceral metastatization. The management of mCRPC has been profoundly modified by introducing novel therapeutic tools such as antiandrogen drugs (i.e., abiraterone acetate and enzalutamide), immunotherapy through sipuleucel-T, and targeted alpha therapy (TAT). This variety of approaches calls for unmet need of biomarkers suitable for patients’ pre-treatment selection and prognostic stratification. In this scenario, imaging with positron emission computed tomography (PET/CT) presents great and still unexplored potential to detect specific molecular and metabolic signatures, some of whom, such as the prostate specific membrane antigen (PSMA), can also be exploited as therapeutic targets, thus combining diagnosis and therapy in the so-called “theranostic” approach. In this review, we performed a web-based and desktop literature research to investigate the prognostic and theranostic potential of several PET imaging probes, such as ^18^F-FDG, ^18^F-choline and ^68^Ga-PSMA-11, also covering the emerging tracers still in a pre-clinical phase (e.g., PARP-inhibitors’ analogs and the radioligands binding to gastrin releasing peptide receptors/GRPR), highlighting their potential for defining personalized care pathways in mCRPC.

## 1. Introduction

Prostate cancer is the second most common cause of death for cancer in developed countries, accounting for 174,650 newly diagnosed cases in USA during 2019 [1]. Localized prostate cancer is treated with radical intent through surgery or radiation therapy, while androgen deprivation therapy (ADT) is used in patients with advanced disease, according to existing guidelines [2]. After a variable time, prostate cancer progresses to the severe hormone-refractory status, also known as “castration-resistant prostate cancer” (CRPC) characterized by a poor prognosis.

CRPC presents a broad spectrum of manifestations, ranging from no symptoms or evidence of metastasis (M0CRPC) to bone and visceral metastatization (mCRPC) with severe debilitation and poor survival [3].

Taxanes-based chemotherapy has represented the upfront treatment for mCRPC for many years [4]. In particular, docetaxel was approved in 2004 by Food and Drug Administration (FDA), since it resulted in prolonging survival in patients affected by mCRPC. Subsequently, another taxane chemotherapeutic, namely carbazitaxel, has been FDA-approved to manage mCRPC patients progressing during docetaxel. The landscape of mCRPC therapy has been deeply modified by introducing and approving second-generation antiandrogens (i.e., abiraterone acetate and enzalutamide) [5,6]. Aside new generation antiandrogens, other treatments have been recently implemented such as poly-(ADP-ribose)-polymerase (PARP) inhibitors (PARPis) which proved to increase survival of mCRPC patients bearing defects in DNA repair genes [7] or immunotherapy with sipuleucel-T, consisting of autologous peripheral blood mononuclear cells, incubated ex vivo with a recombinant fusion protein made of prostate acid phosphate (PAP) to elicit an immune response against prostate cancer cells [8].

After the results of the registrative phase III clinical trial ALSYMPCA, targeted alpha therapy with the bone-seeking radiopharmaceutical radium-223 dichloride (^223^Ra-dichloride) has been FDA-approved for the therapy of bone metastases from mCRPC [9]. Furthermore, prostate specific membrane antigen (PSMA) has emerged as a relevant biomarker, overexpressed in prostate cancer [10]. This variety of therapeutic approaches calls for an unmet need of imaging and laboratory biomarkers suitable for identifying patients who can benefit from a specific treatment to build personalized care pathways. In this scenario, nuclear medicine provides the unique opportunity of exploiting imaging agents, such as ^18^F-FDG and ^18^F-choline, suitable for the characterization of tumor behavior and biological heterogeneity, thus being useful for pre-treatment patients’ prognostic stratification.

Furthermore, some molecular signatures, detected through nuclear medicine techniques, can be combined for diagnosis and therapy in a unique, synergistic approach termed “theranostics” [11]. Theranostics entails “a paradigm shift” with respect to traditional medicine, in which the same therapy is often utilized for different subjects, moving to a more “patient-centered” vision, thus resulting strictly linked to the concept of “personalized medicine”.

One of the most crucial features of theranostics consists of utilizing coupled radiopharmaceuticals with identical or similar properties: the molecule labeled with a diagnostic radionuclide is used for imaging, while the same molecule or a biosimilar agent, labeled with a therapeutic radionuclide, is utilized for therapy. The diagnostic assessment can be performed through a radiopharmaceutical conjugated with a gamma-emitting radionuclide, allowing detection with gamma-camera and single photon emission computed tomography (SPECT), or with a positron-emitter, thus enabling detection through positron emission computed tomography (PET/CT). PET/CT is usually preferred to SPECT for its superior spatial resolution and since it provides the possibility to carry out several quantitative parameters. The diagnostic phase of the theranostic process is mainly aimed to identify if a specific target is present [11]. Furthermore, the diagnostic radiopharmaceutical can be utilized for the in vivo assessment of tracer biodistribution thus providing essential information for the previsional dosimetry [12].

As far as it concerns the therapeutic phase, a compound, with identical or similar characteristics to that utilized for diagnosis is labeled with a radionuclide emitting beta or alpha particles to exert a cytotoxic effect on cells over-expressing the target biomarker. Several theranostic applications currently utilize beta-emitting radionuclides, characterized by relatively long-range through matter and low-energy (linear energy transfer/LET), delivering a cytotoxic effect mainly by determining the formation of oxygen radicals and causing single-strand DNA breaks. Novel theranostic approaches are implementing alpha-emitters, characterized by high LET, very short range in matter, and capable of producing double-strand breaks [13].

In nuclear medicine, an emblematic example of the theranostic approach has been represented by the use of DOTA-labeled peptides binding to somatostatin receptors (SRs) overexpressed by neuroendocrine tumors. In such a case, the specific molecular signature (i.e., SRs) detected by PET/CT with ^68^Ga-DOTA-peptides represents the target for the peptide receptor radionuclide therapy (PRRT) by utilizing beta-emitting ^177^Lu-DOTATATE [14,15].

The aim of the present paper is to review the prognostic and theranostic applications of PET/CT in the field of mCRPC, also underlining the potential of those radiopharmaceuticals that are still in a pre-clinical phase as shown in Table 1.

## 2. Potential Role of PET Tracers as Predictive Biomarkers of mCRPC Response to Therapy

### 2.1. Radiolabeled Choline (^11^C-Choline, ^18^F-Methyl-Choline, ^18^F-Ethyl-Choline)

Choline is the fundamental precursor for the synthesis of phosphatidylcholine, which is the essential component of the mammals’ cell membrane. Once incorporated into the cell, choline is phophorylated by choline-kinase into phosphocholine. It has been demonstrated that choline kinase is over-expressed in malignant cells and also that more aggressive tumors contain large amounts of phospholipids, particularly phosphatidylcholine [54,55].

The use of radiolabeled ^11^C-choline for prostate cancer imaging dates back to 1998, with the first reports from Hara et al. [56]. Nevertheless, the short half-life of the radionuclide ^11^C limited the use of the radiocompound only to clinical center with an on-site cyclotron. A fundamental turning-point for PET imaging with radiolabeled choline has been represented by the synthesis of choline-derivatives labeled with the positron-emitting ^18^F, characterized by a longer half-life. Two ^18^F-labeled derivative radiocompounds, namely ^18^F-methyl-choline (^18^F-cho) and ^18^F-ethyl-choline (^18^F-echo), are currently used in clinical practice. PET/CT with radiolabeled choline is a well-established diagnostic approach for the diagnosis of recurrent prostate cancer after surgery/radiotherapy [56].

As far as it concerns the applications of PET with choline in mCRPC, literature data are still limited. Preliminary reports suggest that several PET-derived parameters might be of value for mCRPC patients’ prognostic stratification before systemic therapy. In particular, PET volumetric parameters, such as Metabolic Active Tumor Volume (MATV) and Total Lesion Activity (TLA), reflecting the overall burden of metabolically active disease, proved to have higher prognostic impact respect to more conventional measurements such as maximum and mean standardized uptake value (SUVmax and SUVmean, respectively) [21,57].

Lee and collaborators measured MATV in 42 patients affected by mCRPC and submitted to PET/CT with ^18^F-cho at baseline and 3 months after the start of therapy (i.e., chemotherapy = 16, antiandrogens = 19, ^223^Ra-dichloride = 5 and sipuleucel-T = 2) [22]. A significant response, defined as a reduction of 30% or greater of MATV, was found in 20 subjects (47.6%). Of note, responding patients according to MATV criteria presented a significantly longer time to PSA progression than non-responders (i.e., 418 days vs. 116 days, *p* = 0.0067). Therefore, the authors suggested that MATV changes, measured with ^18^F-cho PET/CT during treatment, might be predictive of PSA progression, resulting in potential usefulness for switching non-responders to more effective therapeutic options.

Caroli et al. evaluated PET/CT’s prognostic impact with ^18^F-cho in 94 mCRPC patients, progressive after docetaxel, submitted to second-generation antiandrogens (i.e., enzalutamide or abiraterone) [23]. All the enrolled subjects underwent PET/CT before the start of antiandrogen therapy and were then evaluated monthly for biochemical response and every 3 months through radiological assessment. The following parameters were carried out from PET: MATV, TLA and SUVmax. Of note, the sum of MTV and TLA of all the lesions resulted significantly correlated with progression free survival (PFS) and overall survival (OS) in univariate analysis, while only TLA correlated with OS. Thus, the aforementioned paper indicates that the overall metabolically active tumor burden, expressed by TLA calculated on ^18^F-cho PET/CT at baseline, may be useful for identifying patients who are more likely to respond to second-generation antiandrogens.

The predictive role of PET-choline derived parameters for mCRPC management has been recently deepened by Filippi and colleagues in mCRPC subjects treated with ^223^Ra-dichloride [24]. Clinical records of 20 subjects were retrospectively reviewed and the following parameters were calculated on baseline PET/CT with ^18^F-cho: number of lesions, SUVmax, SUVmean, peak of standardized uptake value corrected for lean body mass (SULpeak), TLA and MATV. Clinical and PET-derived parameters were analyzed for prognostic impact on OS. At Kaplan-Meier analysis, baseline PSA levels, number of lesions and TLA resulted significantly correlated with OS, but only TLA remained a significant predictor in multivariate Cox analysis.

The aforementioned papers suggest that volumetric PET parameters, especially TLA, may play an important role in optimizing mCRPC patients’ selection before treatment, thus helping to define personalized pathways of care.

### 2.2. ^18^F-fluorodeoxyglucose (^18^F-FDG)

PET/CT with ^18^F-FDG represents a widely used imaging method for diagnosis and follow-up of the majority of malignancies. This diagnostic approach’s clinical success relies on the fact that most of the tumors utilizes anaerobic glycolysis to produce energy, even in the presence of functioning mitochondria. This phenomenon, also known as “Warburg effect”, entails an increased incorporation of glucose, and consequently also of ^18^F-FDG, in malignant tissue through the overexpression of glucose-transporters (GLUT), especially the subtype 1 (GLUT-1) [58].

It is well known that prostate cancer, especially the more differentiated forms, do not exhibit a relevant Warburg effect, thus being characterized by absent or low ^18^F-FDG avidity. Nevertheless, when progressing to the state of mCRPC, prostate tumors switch to glycolysis as a preferential pathway for producing energy [59].

Several types of research have been made to investigate the prognostic implication of ^18^F-FDG uptake in mCRPC. Fox and collaborators investigated the prognostic impact of dual tracer PET/CT with ^18^F-FDG and ^18^fluorodihydrotestosterone (^18^F-FDHT) for determining the androgen receptor (AR) and glycolytic (Gly) status in 133 mCRPC patients submitted to therapy with second-generation antiandrogens [25]. All the enrolled subjects, affected by mCRPC progressive after ADT or ADT/chemotherapy, performed both PET examinations within an interval of time ranging 12–29 days and, in every case, before the start of therapy with enzalutamide or abiraterone acetate. On the basis of PET/CT examination, patients were categorized in 4 groups: (1) patients bearing lesions all avid for both tracers (AR+/Gly+); (2) those having concordant lesions but with at least 1 localization positive at ^18^F-FDHT and negative at ^18^F-FDG (AR+/Gly0); (3) those having concordant lesions but with at least 1 localization negative at ^18^F-FDHT and positive at ^18^F-FDG (AR0/Gly+); (4) subjects with a mixture of concordant lesions. The different imaging phenotypes detected by dual tracer PET/CT were correlated with patients’ outcome after antiandrogen therapy. On a patient-basis analysis, imaging phenotypes belonging to groups 3 and 4 had the most negative effect on survival, suggesting that the glycolytic pathway’s activation might be linked to resistance to second-generation anti-androgens.

More recently, Bauckneht et al. evaluated the prognostic role of PET/CT with ^18^F-FDG in 28 mCRPC patients submitted to therapy with ^223^Ra-dichloride: in all the enrolled subjects, the following parameters were carried out: SUVmax of the hottest bone lesion, metabolic tumor volume (MTV) and total lesion glycolysis (TLG) [26]. Furthermore, 20 out of 28 patients underwent a post-therapy PET/CT and response to ^223^Ra-dichloride was assessed according to PET Response Criteria in Solid Tumors (PERCIST). Among the analyzed baseline PET and clinical parameters, baseline PSA, LDH, and MTV significantly correlated with OS. Furthermore, MTV resulted useful to identify a subgroup of patients with a worse prognosis. As far as it concerns the value of ^18^FDG PET for monitoring response to targeted alpha therapy, subjects showing partial metabolic response had longer survival respect those with stable or progressive disease.

### 2.3. Androgen Receptor (AR)

AR, codified by a gene located in chromosome X, is a 11 kDa protein consisting of 4 regions: a NH2 terminal transactivation domain (NTD), a DNA-binding domain (DBD), a hinge region and a ligand binding domain (LBD). Both testosterone and dehydrotestosterone (DHT) bind to the LBD of AR, thus inducing a conformational change in AR, which translocates into the nucleus, creates a dimer and then binds to a specific region of DNA through the DBD [60]. AR plays a crucial role in prostate cancer growth and development. Additionally, in the state of mCRPC, it has been demonstrated that tumor proliferation and spreading is still driven by AR, through several and still not fully understood mechanisms, among whom AR gene amplification, entailing an overexpression of AR, has been found in 30% to 50% of CRPC patients. The considerations above led to the development of 2nd generation antiandrogens (i.e., abiraterone acetate and enzalutamide), which proved useful to increase patients’ lifespan, prolonging metastasis-free overall survival, and reducing serum PSA levels [5]. The choice of submitting mCRPC patients to antiandrogens-based therapy, rather than to chemotherapy or immunotherapy with sipuleucel T, mainly relies on the entity of the overall disease burden and on the presence and severity of symptoms.

However, an imaging method capable of addressing the individual biological sensitivity to antiandrogens would be much welcome for a patient-tailored pre-treatment stratification.

On this path, efforts have been devoted to the development of a steroid-based radiocompound, characterized by an affinity for AR and suitable for in vivo determination of AR status. The radioligand ^18^fluorodihydrotestosterone (^18^F-FDHT) resulted in presenting a high affinity for target, adequate stability in vivo and in vitro, a relatively easy process synthesis [61]. After i.v. administration, the tracer binds to serum protein, preferentially to the sex hormone–binding globulin (SHBG); subsequently, it is transferred to the destination cellular compartment through intramolecular transport and binding to AR.

In a preliminary report by Dehdashti et al. on 20 patients with advanced prostate cancer, PET/CT scan with ^18^F-FDHT detected AR-positive lesions in 12 patients who were submitted to a repeated PET/CT after the administration of an AR antagonist (i.e., flutamide): in all cases, a decrease in ^18^F-FDHT uptake, measured both as SUV and tumor-to-muscle ratio (T/M), was registered, thus suggesting the incorporation of ^18^F-FDHT being a receptor-mediated process [33]. Patients’ selection before PET/CT with ^18^F-FDHT is of utmost importance since binding the tracer to AR is feasible only in subjects with castrate levels of androgens. It is worth mentioning that AR imaging by ^18^F-FDHT was applied in the clinical trial assessing the safety and antitumor activity of second-generation antiandrogen enzalutamide [34] and apalutamide [62].

One of the crucial technical aspects for determining PET/CT’s potential with ^18^F-FDHT for monitoring AR-status in mCRPC patients undergoing hormonal treatments is represented by its high inter-observer reproducibility (ICC) and repeatability. In a prospective, multicenter study including 27 subjects submitted to 2 ^18^F-FDHT PET/CT in 2 consecutive day (test/rest modality), no significant differences were registered as concerns PET-derived parameters (i.e., SUVmax, SUVmean, and SUVpeak) with an ICC > 0.98 [35].

## 3. Theranostic PET Tracers

### 3.1. Bone-Targeting Theranostic Radiopharmaceuticals

#### 3.1.1. ^18^F-Sodium Fluoride (^18^F-NaF)

Recently introduced target alpha therapy (TAT) through ^223^Ra-dichloride has triggered imaging agents’ research suitable for patients’ selection. The radiopharmaceutical ^223^Ra-dichloride is incorporated into forming-bones through a not completely understood mechanism, mainly based on the similarity between radium and calcium, the latter being the essential component of hydroxyapatite [63].

First efforts to carry out dosimetric calculations for ^223^Ra-dichloride have been made by Pacilio et al., exploiting its similarity with 99mtechnetium-labeled diphosphonates (^99m^Tc-MDP), the tracer commonly used in clinical practice for bone scintigraphy [64]. The authors analyzed 9 patients with 24 skeletal metastases from mCRPC, submitted to 6 cycles every 4 weeks consisting in the administration of 50 kBq/Kg of ^223^Ra-dichloride. Patients were imaged with gamma-camera on ^223^Ra gamma-photopeak for 30 min after radiopharmaceutical injection. Lesions were contoured on ^99m^Tc-MDP whole body images and then superimposed on ^223^Ra-images after co-registration. The authors demonstrated the feasibility of an in vivo quantification of absorbed dose-to-lesion in ^223^Ra-therapy and found a correlation between ^99m^Tc-MDP uptake and the incorporation of ^223^Ra-dichloride.

In light of the above, several researchers have investigated the potential of the PET tracer ^18^F-NaF, namely ^18^F-fluoride, as a theranostic agent for selecting patients before TAT with ^223^Ra-dichloride, since ^18^NaF is a bone-seeking agent, with a mechanism of incorporation similar to that of ^99m^Tc-MDP and ^223^Ra-dichloride [65].

Blau and colleagues firstly described the kinetics of ^18^F-NaF [66], indicating that the ion ^18^F needs to pass from plasma to extracellular space and then into hydroxyapatite crystal through a chemo-absorption process. Once absorbed, the ion ^18^F exchanges with the group −OH and forms fluoroapatite. Therefore, the accumulation of ^18^F-NaF has been investigated as a surrogate biomarker of altered bone metabolism “turn-over” in animal models before its introduction in clinical practice. PET/CT with ^18^F-fluoride has been proved extremely sensitive for the detection of skeletal metastases. Araz and collaborators compared the diagnostic performance of PET/CT with ^18^F-NaF and scintigraphy with ^99m^Tc-MDP in 37 patients with bone metastases: PET/CT with ^18^F-NaF outperformed scintigraphy in the 89% of the enrolled patients, showing a significantly greater number of localizations, and revealing both lytic and osteoblastic metastases [67].

Since ^18^F-NaF and ^223^Ra-dichloride share hydroxyapatite as common target, it has been hypothesized that PET/CT with ^18^F-NaF might be applied for predicting bone lesions’ response to TAT. In a phase I open-label clinical trial, five patients affected by mCRPC were submitted to ^99m^Tc-MDP scintigraphy and PET/CT with ^18^F-NaF before the 1st administration of 100 kBq/kg (therapy 1) and then also immediately before the 2nd administration, performed 6 weeks later (therapy 2) [16]. On a lesion-based analysis, the authors demonstrated correlation between absorbed dose-to-lesion and serial changes in ^18^F-NaF uptake and between ^18^F-NaF uptake and ^223^Ra-incoporation. Although needing confirmation with larger cohorts, the aforementioned previous results suggested that PET/CT with ^18^F-NaF might be useful to predict patients’ response to ^223^Ra-therapy and optimize radiopharmaceutical delivery through personalized dosimetry.

Another interesting research performed by Kairemo and coworkers investigated ^18^F-NaF PET/CT’s potential usefulness for assessing the response to ^223^Ra-therapy [17]. In 10 mCRPC patients with skeletal lesions treated with 6 cycles of ^223^Ra-dichloride, PET/CT with ^18^F-NaF was performed at baseline, after the 1st and the 6th cycles and a modified version of the PET Response Evaluation Criteria (PERCIST) was utilized for determining the response to treatment. The authors demonstrated that the metabolic response assessed after the 6th cycle was correlated with the biochemical response (i.e., PSA reduction) in 9/10 patients.

#### 3.1.2. Radiolabeled Zolendronic Acid

Biphosphonates are widely used in clinical practice for the management of metastatic skeletal disease, especially for pain palliation, since these compounds strongly bind to hydroxyapatite inhibiting its aggregation and dissolution.^99m^Tc-methylene diphosphonate is routinely utilized in the work-flow of PCa while samarium-153 (^153^Sm) ethylene-diamine-tetra-methylene phosphonate has been successfully applied for bone palliation due to its incorporation in areas with increased osteoblastic activity.

In recent years, zoledronic acid has emerged as one of the most effective last-generation bisphosphonates, since it is characterized by very high hydroxyapatite affinity and results capable to inhibit the farnesyl diphosphate synthase [68]. This evidence triggered the development of the DOTA-conjugated zolendronic acid, namely DOTA^ZOL^, suitable for the labeling with several radiometals either for imaging (i.e., ^68^Ga) or therapeutic (i.e., ^177^Lu) purposes.

In a preliminary report, PET/CT with ^68^Ga-DOTA^ZOL^, carried out in a patient with mCRPC, demonstrated highly increased tracer incorporation in skeletal lesions, consistent with the findings of contextually performed ^68^Ga-PSMA-11 PET/CT [69].

Khawar and colleagues evaluated ^68^Ga-DOTA^ZOL^’s biodistribution in 5 patients with metastatic skeletal disease (i.e., breast cancer, bronchial carcinoma and mCRPC) and found high tracer uptake in bones as target organ, kidneys and urinary bladder as organs of excretion and faint uptake in liver, spleen and salivary glands [18]. The authors suggested that, by means proper hydration and diuresis in order to minimize urinary retention and irradiation, ^68^Ga-DOTA^ZOL^ might represent an alternative to ^18^F-NAF for the PET imaging of metastatic skeletal disease, also being suitable for carrying out a personalized provisional dosimetry in the perspective of a radionuclide treatment with ^177^Lu-DOTA^ZOL^.

In a research from Khawar et al., the biodistribution and dosimetry of ^177^Lu-DOTA^ZOL^ was assessed in a group of 4 patients with skeletal metastases from bronchial carcinoma or mCRPC, treated with a mean dose of 5968 ± 64 MBq [19]. Quantitative analysis demonstrated high mean residence time of the tracer in skeleton and kidneys, with the highest absorbed dose in osteogenic cells, kidneys and bladder walls. Worthy of note, the authors found that the dose delivered to kidneys was inferior for ^177^Lu-DOTA^ZOL^ respect to that of ^177^Lu-PSMA-617.

These preliminary data were further confirmed by Fernandez and collaborators, who evaluated the safety and dosimetry of a single dose administration of ^177^Lu-DOTA^ZOL^ in 9 patients with bone metastases from mCRPC [20]. The authors found a favorable therapeutic index for the radiocompound, with average absorbed doses in skeletal metastases, kidneys, red bone marrow, and bone surfaces of 4.21, 0.17, 0.36 and 1.19 Gy/GBq, respectively. Although promising, the aforementioned results need to be further supported by well-designed, randomized clinical trials.

### 3.2. Molecular Theranostic Radiopharmaceuticals

#### 3.2.1. Prostate Specific Membrane Antigen (PSMA)

**Figure 1:**
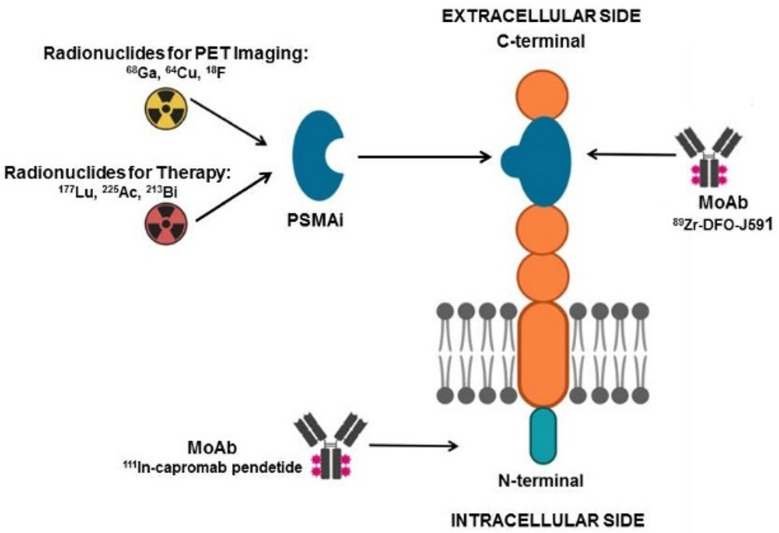
Schematic representation of PSMA molecular structure. PSMA inhibitors (PSMAi) expolit glutamate carboxypeptidase enzymatic activity of the extracellular domain of PSMA and can be labeled either with positron-emitting nuclides (e.g., ^68^Ga, ^64^Cu, ^18^F) for imaging or with beta/alpha emitting radioisotopes (e.g., ^177^Lu, ^213^Bi, ^225^Ac) for radionuclide therapy. Monoclonal antibodies (MoAbs) have also been developed: ^111^In-capromab is directed towards the intracellular PSMA domain and is utilized for scintigraphic imaging, ^89^Zr-DFO-J591 targets the extracellular portion of PSMA and is suitable for PET imaging (figure created with Biorender.com (accessed on 8 March 2021)).

In recent years, PSMA has emerged as one of the most attractive theranostic targets for prostate cancer. It is a type II, 750 amino-acid transmembrane protein, characterized by an enzymatic glutamate carboxypeptidase activity. PSMA is codified by a gene located in the short arm of the chromosome 11, usually non deleted in prostate cancer, and is constituted by a 3-part structure: a 19-amino-acid internal portion, a 24-amino-acid transmembrane portion, and a 707-amino-acid external portion [70]. Figure 1 schematizes PSMA structure and the potential theranostic approaches directed towards PSMA.

The exact role of PSMA in normal prostate tissue has still to be defined yet, but it has been hypothesized that it might have a transport function since, after interaction with PSMA, PSMA-ligands are internalized through endocytosis [71].

The important role of PSMA as target for diagnosis and therapy of prostate cancer relies on its overexpression in prostate adenocarcinoma while it is only weakly detectable in normal prostate tissue. Immunohistochemical studies demonstrated that PSMA staining can be revealed in the 94.1% of cancers and results significantly associated with tumor stage, high Gleason grade and preoperative PSA [72]. However, it has to be underlined that PSMA overexpression can also be found in non-pathological and non-prostatic tissue such as salivary glands, the duodenal mucosa, proximal renal tubular cells, and neuroendocrine cells in the colonic crypts [73].

First efforts of developing a PSMA-targeting tracer date back to the end of the 80s, when the monoclonal antibody (MoAb) 7E11 was synthesized and conjugated with the gamma-emitting radionuclide indium-111 (^111^In). The radioimmunoconiugate ^111^In-7E11, namely indium ^111^In-capromab pendetide (ProstaScint^®^), was approved by FDA in 1996 and introduced in clinical practice for the imaging of prostate cancer through gamma-camera and SPECT [74]. Its capability of binding represents the main limitation of 111In-capromab pendetide only to the intra-cytoplasmatic portion of PSMA, which is normally expressed by damaged and dying tumor cells. A further issue is linked to the technology utilized for detection, since SPECT has a limited spatial resolution and does not allow quantitative calculations, although the hybrid SPECT/CT modality proved useful to improve the accuracy of conventional scintigraphic imaging [75,76].

Another interesting approach for PSMA-targeting imaging was provided by the development and synthesis of J591, a MoAb binding to the extracellular portion of the PSMA structure. J591 MoAb was firstly conjugated with the gamma-emitting ^111^In and, subsequently, with the positron-emitter zirconium-89 (^89^Zr). Holland et al. reported first pre-clinical data on using the MoAb J591, conjugated with ^89^Zr through the chelator desferrioxamine (DFO) [27]. The radiocompound, namely ^89^Zr-DFO-J591, was tested first in vitro and then in 2 groups of nude mice bearing sub-cutaneous LNCaP (PSMA positive) or PC-3 (PSMA negative) tumor xenografts. The authors found that ^89^Zr-DFO-J591 bound to PSMA-expressing tumor cells with an immunoreactive fraction of 0.95 ± 0.03. Furthermore, micro-PET examination showed that ^89^Zr-DFO-J591 was capable to detect PSMA-positive xenografts (i.e., LNCaP) with tumor-to-background > 20 between 48–144 h post-injection. This approach, termed immuno-PET, provides several advantages, since it combines the high specificity of MoAb-based detection with the advanced technological performance of PET/CT scan. Nevertheless, immuno-PET with ^89^Zr-DFO-J591 has not passed to the clinical phase yet, mainly due to issues linked to ^89^Zr-DFO-J591 immunogenicity and its slow clearance from circulation.

A turning-point in the field of PSMA imaging has been represented by the synthesis of small PSMA inhibitors consisting of compounds linked to glutamate isostere or glutamate, which binds to PSMA extracellular domain exploiting its enzymatic activity [77]. In particular, the radiopharmaceutical ^68^Ga-PSMA-HBED-CC, namely ^68^Ga-PSMA-11, proved useful for the in vivo imaging of prostate cancer and its metastatic localizations [78] and has been recently approved by FDA (https://www.accessdata.fda.gov/drugsatfda_docs/label/2020/212642s000lbl.pdf, accessed on 8 March 2021).

The strongest evidences of the clinical impact of PET/CT with ^68^Ga-PSMA-11 in PCa patients’ management have been provided by the recently published prospective randomized clinical trial proPSMA [28]. A cohort of 302 men affected by high-risk PCa were randomly assigned to conventional imaging (i.e., CT + bone scan) or to PET/CT with ^68^Ga-PSMA-11 as first-line imaging approach. PSMA PET/CT showed a 27% greater accuracy than conventional imaging, especially as far as it concerns the detection of pelvic lymph node metastases, leading to patients’ management change in the 28% of the examined subjects.

As far as it concerns the role of PET/CT with ^68^Ga-PSMA-11 in mCRPC, scientific data are still limited. In a recently published meta-analysis, PET/CT with ^68^Ga-PSMA-11 presented higher sensitivity for detecting bone lesions than conventional bone scintigraphy and upstaged patients from the state of non-metastatic CRPC to that of mCRPC, with consequent impact on therapeutic management [79]. In this regard, Fourquet et al. retrospectively investigated PET/CT’s clinical impact with ^68^Ga-PSMA-11 in 30 non-metastatic and asymptomatic patients with rise in PSA despite a suitable castrate serum level of testosterone. The detection rate of PSMA PET/CT resulted in 90%, significantly higher in patients with PSA greater than 2 ng/mL (i.e., 100%) than in those with PSA < 2 ng/mL (i.e., 70%) [80]. Of note, the results of PSMA PET meaningfully changed patients’ management in the 70% of cases, in particular, 14 subjects were submitted to second-generation antiandrogens and 1 underwent chemotherapy with taxanes.

Aside its relevant role in diagnostics, the theranostic potential of PSMA-ligands relies on the possibility of substituting the positron-emitter with a radionuclide emitting beta- (i.e., ittrium-90/^90^Y or luthetium-177/^177^Lu) or alpha particles (i.e., actinium-225/^225^Ac or bismuth-213/^213^Bi).

For radioligand therapy (RLT) with ^177^Lu, two radiopharmaceuticals have been developed: ^177^Lu-PSMA-617 [81] and ^177^Lu-PSMA I&T (imaging and therapy) [82]. FDA or EMA has not approved PSMA-based RLT, therefore its use has been limited to clinical trials enrolling mCRPC patients progressing during approved treatments. In the majority of the clinical studies, administered activity per cycle ranged 6–7.4 GBq for an overall number of 4–6 cycles, the most relevant adverse effect was represented by xerostomia, since PSMA inhibitor is physiologically incorporated into salivary glands [83].

**Figure 2:**
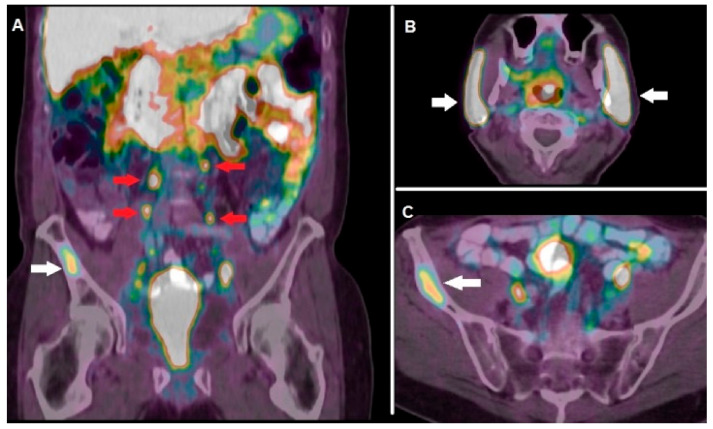
A 74-year-old patient affected by mCRPC (Gleason score 5 + 4), progressing after surgery, androgen deprivation therapy (ADT) and chemotherapy, submitted to PET/CT with ^68^Ga-PSMA-11 before enrollment for ^177^Lu-RLT. Coronal fused images (**A**) showed highly increased tracer incorporation in abdominal lymph nodes (red arrows) and in the right iliac bone (white arrow). Note the intense physiological radiopharmaceutical uptake in salivary glands (**B**), white arrows), while fused axial image of the pelvis well demonstrates ^68^Ga-PSMA-11 uptake in the iliac metastasis (**C**) white arrow).

In a multicentric study encompassing 99 mCRPC patients submitted to ^177^Lu-PSMA-617 RLT, 45 subjects (i.e., 45%) presented a PSA decline ≥ 50% over the follow-up period and were therefore considered as responders [84]. However, complete response after ^177^Lu-PSMA RLT is rarely reported and relapse often occurs. These evidences triggered the research of radiocompounds suitable for a PSMA-targeted alpha therapy, thus exploiting the more favorable radiobiological properties of alpha-emitters. Preliminary clinical experiences with PSMA-617 conjugated with the alpha emitters ^225^Ac and ^213^Bi report a high rate of response (i.e., 65–70%) being salivary glands’ toxicity the dose-limiting- factor [85]. Figure 2 shows a clinical case of a patient affected by mCRPC, evaluated with ^68^Ga-PSMA-11 before enrollment for RLT.

Pre-treatment patients’ selection and stratification represent a crucial issue for PSMA-based RLT. In this regard, pre-therapeutic assessment of the grade of PSMA-expression through PET/CT with ^68^Ga-PSMA-11 has been found of utmost importance. For example, at least 1 site of a metastatic disease characterized by significantly greater uptake than normal liver on baseline ^68^Ga-PSMA-11 PET/CT was established as enrollment criterion for ^177^Lu-PSMA-617 RLT in the single-arm, single-center, phase 2 trial aimed to assess treatment safety, efficacy, and impact on quality of life in mCRPC patients [86]. Of note, all the included patients also underwent a pre-treatment ^18^FDG PET/CT in order to exclude the presence of ^18^FDG positive/PSMA harmful sites of disease.

A recently published retrospective study further assessed the prognostic impact of PSMA expression, assessed on pre-treatment PET/CT with ^68^Ga-PSMA-11, on survival after ^177^Lu-PSMA-617 RLT in 85 subjects affected by mCRPC [29]. The authors calculated several quantitative parameters, among whom average SUVmax of all metastases (PSMA_average_), that resulted a significant prognosticator of OS, while subjects with low PSA expression presented a worse prognosis. These interesting preliminary data support ^68^Ga-PSMA-11 PET/CT’s mandatory role for subjects’ selection before a PSMA-based RLT, although further studies are needed to define if the average grade of PSMA-expression, or rather the number of PSMA-expressing metastases, can have a major predictive role.

Despite its growing application in diagnostics, ^68^Ga-PSMA-11 presents some limitations, such as the suboptimal energetic resolution, the need of an on-site generator for ^68^Ga-production and the relatively short half-life of ^68^Ga (i.e., 68 min).

Alternatively to PET/CT with ^68^Ga-PSMA-11, other radionuclides have been applied for the labeling of PSMA-inhibitors. In particular, preliminary reports indicate the clinical usefulness of PSMA-ligand conjugated with the radionuclide copper-64 (^64^Cu). The isotope ^64^Cu presents particularly favorable physical properties, since it emits both positrons and electrons (i.e., maximum energy of 0.58 MeV). This characteristic makes ^64^Cu a real theranostic agent, since it can be used, at different dosages for diagnosis or therapy. As specifically concerns ^64^Cu-PSMA, scientific data are still preliminary and not explicitly focused on mCRPC [87].

Several efforts have been made to synthesize radiocompounds binding to PSMA and labeled with the radionuclide ^18^F, which presents optimal energetic resolution for PET/CT detection and sufficiently long half-life such as to enable radiopharmaceutical delivery to clinical centers distant from the production site. Bouvet and colleagues developed the radiocompound ^18^F-DCFPyL, via direct radiofluorination, validated in a pre-clinical model, consisting of nude mice bearing LNCaP (PSMA+) and PC3 (PSMA−) tumor xenografts [30]. Dynamic PET was utilized for investigating ^18^F-DCFPyL biding to tumor xenografts in both mice groups: high and rapid tracer incorporation was detected in LNCaP (i.e., PSMA+) xenografts, increasing over time and achieving a tumor-to-blood pool ratio of 8.3 at 60 min. On the contrary, continuous wash out of the radio compound was registered in the PSMA—xenografts (i.e., PC3).

Information regarding the application of PET/CT with ^18^F-DCFPyL in clinical practice are still limited. In a recently published study including 160 patients with high-risk prostate cancer, PET/CT with ^18^F-DCFPyL at staging detected metastatic localizations in 90% of subjects (M1a, M1b and, M1c in 49%, 28%, 31% and 3% respectively), revealing lymph node metastases in 41/42 patients and impacting on patients’ clinical management in 17% of cases [88].

As regards the specific condition of mCRPC, it has been reported that PET/CT with ^18^F-DCFPyL reveals an increased PSMA expression in mCRPC patients submitted to second-generation antiandrogens, evaluated 3 months after the start of therapy, most probably due to PSMA-overexpression consequent to treatment-induced androgen receptors downregulation [89].

As far as it concerns PSMA-targeted theranostics, it is worth mentioning the interesting emerging role of the group of radionuclides belonging to the Terbium (Tb) group. Some Tb radioisotopes, in fact, such as ^152^Tb and ^155^Tb, have physical characteristics suitable for PET or SPECT imaging, while ^149^Tb and ^161^Tb have energetic features favorable for therapy, since they are alpha and Auger emitters, respectively [31]. Müller et al. successfully applied ^152^Tb-PSMA-617 for the PET/CT visualization of PSMA-expressing tumors in mice and also used the aforementioned tracer in a mCRPC patient, obtaining images of diagnostic quality. On this path, the same group of research investigated the potential of ^161^Tb-labeled PSMA as a theranostic agent in mCRPC [32]. The authors, in fact, utilized ^161^Tb-PSMA-617 both for SPECT imaging, by virtue of its gamma-emission, and for achieving an anti-tumoral effects, due to its abundant co-emission of conversion and Auger electrons, in mice bearing PSMA-positive tumor xenografts. These preliminary results suggest that ^161^Tb-PSMA-617 might represent an effective alternative to ^177^Lu-PSMA-617 for RLT.

#### 3.2.2. Poly (ADP-Ribose) Polymerase-1 (PARP-1)

Poly (ADP-Ribose) polymerase-1 (PARP-1) protein plays a key role in DNA damage repair and transcriptional regulation. Interaction with damaged DNA activates poly-ADP-ribosylation reaction that is a reversible post-translational modification of proteins resulting in the covalent attachment of a polymer of ADP-ribose units on a variety of amino acids residues on target proteins [90].

PARP1 is overexpressed in several types of cancer such as ovarian, breast, oral, and prostate cancer. As specifically regards prostate cancer, PARP 1 plays many roles such as mediating DNA damage repair, transcriptional regulation, and nuclear hormone receptor signaling, and can be an optimal target for non-invasive imaging and therapy as several studies demonstrated.

In recent years a new therapy based on the use of PARP inhibitors (PARPis) was developed. Jang et al. analyzed the mechanism of action and rationale for the use of PARPis, alone or in combination with other therapies especially in patients with metastatic castration-resistant PCa (mCRPC) [91]. The previously cited review evaluated several studies based on the use of two types of PARPis, Olaparib, and Rucaparib, which have produced encouraging results.

While PARP-1 targeted inhibitors have been relatively fast developed and approved, the determination of PARP-1 expression might help to predict the response to PARP inhibitor treatment. Sankaranarayanan et al. summarize the recent preclinical advancements in imaging and theranostic PARP-1 targeted tracers [92]. To estimate PARP1 levels, several imaging probes with fluorescent or gamma/beta-emitting radionuclides have been proposed.

In particular, ^18^F-conjugated PARPis-like Olaparib (^18^F-Olaparib), olaparib derivatives and rucaparib derivatives (^18^F-FT, ^18^F-WC-DZ-F) have been evaluated as potential tools for the in vivo determination of PARP-1 expression. Especially ^18^F-WC-DZ-F, a rucaparib derivative, has been characterized in a subcutaneous prostate cancer model and was developed by replacing the radionuclide ^125^I in the ^125^I-KX1 molecule with ^18^F to improve pharmacokinetics in vivo tumor uptake and enhance blood stability.

Zhou et al. showed the potentiality of ^18^F-WC-DW-F. In microPET imaging study ^18^F-WC-DW-F accumulated and remained in the target tumor (close to 4% ID/c.c. at 2-h post-injection) but readily washed out from other organs and tissue, except for the liver [36].

Theranostic PARP can be a useful tool for analyzing tumor characteristics and predicting the response to therapy. At the same time, the overexpression of PARP1 can afford the possibility to defeat tumor with greater specificity. Furthermore, several efforts have been made to develop PARP-radioligands suitable to inflict DNA damage on cancer cells, in particular through the so-called “Auger emission”, consisting by low-energy electrons released by radionuclides decay by electron capture [93]. Since PARP-1 represent an abundant nuclear enzyme, Auger-emitting PARP-radioligands exhibit a relevant potential to deliver a cytotoxic effect to cancer cell DNA. Preliminary reports utilizing several Auger-emitting theranostic tracers, such as ^123^I-MAPi (Iodine-123 Meitner-Auger PARP1 inhibitor) and the already cited ^125^I-KX1 provided encouraging results in tumors other than prostate cancer, such as glioblastoma multiforme and neuroblastoma [94]. Nevertheless, to the best of our knowledge, this theranostic approach has not been applied explicitly to the mCRPC model.

#### 3.2.3. Gastrin Releasing Peptide Receptor (GRPR)

Gastrin release peptide receptors (GRPRs), also known as BB2 receptors, are G-protein coupled receptors that are expressed in many human tissues, such as pancreas, stomach, adrenal cortex and brain. They belong to the bombesin receptor family, along with neuromedin B receptor (NMBR, BB1) and the bombesin receptor subtype-3 (BRS-3, BB3). The overexpression of these receptors in several human cancers, including prostate, breast, and pancreatic cancer, makes GRPRs a potential molecular target for diagnostic and therapeutic purposes. In the last two decades, many studies have focused on the development of radiolabelled bombesin-like peptides for PET/CT imaging and treatment of prostate cancer.

The first studies regarding the use of GRPRs for PET/CT imaging and treatment of prostate cancer were based on radiolabelled GRPR-agonists, such as ^68^Ga-AMBA/^177^Lu-AMBA. ^68^Ga-AMBA showed, in mice xenografted with 22Rv1 prostate cancer cell line, good tumor uptake, and a high tumor-to-background ratio according to Zhang-Yin et al. [37]. In another study ^55^Co-NOTA-AMBA in PC3 xenografted mice was superior for PET/CT imaging, compared to ^68^Ga-NOTA-AMBA since it showed a better tumor-to-organ ratio [38]. In 2009, a study from Maddalena et al. showed the good therapeutic potential of ^177^Lu-AMBA in LNCaP, DU145, or PC-3 tumor-bearing male nude mice [39].

In 2007, Zhang et al. developed DOTA-PESIN, a novel DOTA-conjugated bombesin derivative, and labeled it with ^67^Ga, ^68^Ga, and ^177^Lu for investigating its use for diagnosis and radionuclide therapy in prostate cancer on xenografted mice bearing PC-3 human prostate tumor [40]. ^67^Ga/^177^Lu-DOTA-PESIN showed high uptake in the human prostate tumor xenografts and in murine GRPR-positive organs, PET images demonstrated that ^68^Ga-DOTA-PESIN accumulates predominantly in the PC-3 tumor, pancreas, and kidneys. ^177^Lu-DOTA-PESIN also displayed a high uptake in the tumor and a relatively slow washout. In conclusion, DOTA-PESIN proved to have high potential concerning PET and SPECT images and targeted radionuclide therapy with ^177^Lu.

Nevertheless, it was shown that such peptides, by binding and activating their receptors, could induce several adverse effects in the gastrointestinal system, therefore the attention on the radiolabeling of GRPR target molecules has rapidly shifted toward the use of GRPR antagonists.

GRPR antagonists have more favorable pharmacokinetics than their agonist counterparts. While GRPR antagonists do not activate the receptor upon binding, they can successfully target and be retained in GRPR-expressing cancer lesions while rapidly clearing from a physiological organ in both animal models and humans [95].

A clinical study performed by Wieser et al. in 2014, investigated the biodistribution, dosimetry, and tumor uptake of the GRPR antagonist ^64^Cu-CB-TE2A-AR06 by PET/CT in 4 patients with newly diagnosed prostate cancer [41]. In 3 out of 4 patients, the prostate tumor was visualized with high contrast on the ^64^Cu-CB-TE2A-AR06 PET scans at all-time points. ^64^Cu-CB-TE2A-AR06 was rapidly accumulated by prostate cancer and cleared much slower from the tumors than from normal organs, suggesting that the high tumor uptake of GRPR ligands and the low intestinal and kidney uptake of GRPR antagonists make these compounds particularly attractive for peptide receptor-targeted radiotherapy (PRRT).

Maina et al. introduced the DOTA-conjugated GRPR antagonist SB3 and labeled it with ^67^Ga or ^68^Ga. At the preclinical level, ^67^Ga-SB3 strongly and bound explicitly onto the membrane of PC-3 cells, showing good stability in the mice’s bloodstream, high uptake of ^67^Ga-SB3 in prostate cancer xenografts and high uptake in the mice pancreas. The results of this preclinical study encouraged the first-in-man study including a small number of disseminated prostate cancer and breast cancer. In the examined patients, the highest physiological uptake of ^68^Ga-SB3 was found in the pancreas head, kidneys, and esophagogastric junction, being positive lesions visualized in 5 of 9 cases (55%) by ^68^Ga-SB3 on PET/CT [42].

This study’s promising findings led to a further evaluation of SB3 radiolabelled with ^111^In for SPECT imaging and ^177^Lu for radionuclide therapy. Lymperis and colleagues compared the bioavailability of ^111^In/^177^Lu-SB3 to that of ^68^Ga-SB3, demonstrating the need for coinjection of the radioconiugates with neprilysin (NEP)-inhibitor phosphoramidon (PA) to enhance radiolabel uptake in PC-3 tumors [43]. The importance of avoiding in vivo degradation by proteolytic enzymes of small peptides was already highlighted by Chatalic et al. [44], according to whom in PC-3 tumor-bearing mice, PA co-injection leads to enhanced PC-3 tumor signal intensity in PET imaging with ^68^Ga-JMV4168, as well as regression of tumor size and increased survival rate following radionuclide therapy with ^177^Lu-JMV4168.

A novel DOTA-coupled GRPR antagonist derived from SB3, NeoBOMB1, was studied by Dalm et al. [45]. The authors evaluated the biodistribution of ^68^Ga-NeoBOMB1 for PET/CT imaging and ^177^Lu-NeoBOMB1, for radionuclide therapy in a mouse model, and reported good visualization of the tumor tissue in PET images and high uptake in PC-3 cells, respectively.

A first in human dosimetry was published by Kurth et al., who enrolled 35 patients with mCRPC without further treatment options submitted to PET/CT with the radiolabeled synthetic bombesin receptor antagonist ^68^Ga-RM2 prior to therapy with the beta-emitting ^177^Lu-RM2 [46]. Among these patients, 4 underwent therapy with, on average, an activity of 4.48 GBq of ^177^Lu-RM2, which showed high tumor uptake and rapid clearance from normal organs. The therapy was well-tolerated by patients, with no relevant side effects. The absorbed doses in tumor lesions (6.20 ± 3.00 mGy/GBq) and metastases were therapeutically relevant. The highest uptake and absorbed dose was found in bone metastases, while the mean absorbed dose to red bone marrow (0.02 ± 0.01 mGy/GBq) was below the critical value, being pancreas the most sensitive and dose-limiting organ (1.08 ± 0.44 mGy/GBq).

**Figure 3:**
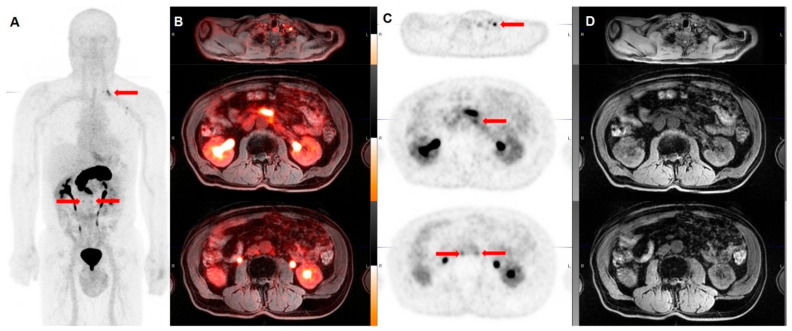
Whole body Maximum Intensity Projection (MIP, **A**), fused coronal ^68^Ga-RM2-PET/MRI (**B**), coronal emissive ^68^Ga-RM2-PET (**C**), and MRI images (**D**) in a 74 year-old man with PCa in biochemical recurrence (PSA 8.4 ng/mL). Focal 68Ga-RM2 uptake is seen in left supraclavicular and retroperitoneal lymph nodes (red arrows). (Images courtesy of Andrei Iagaru, MD, Stanford University).

**Figure 4:**
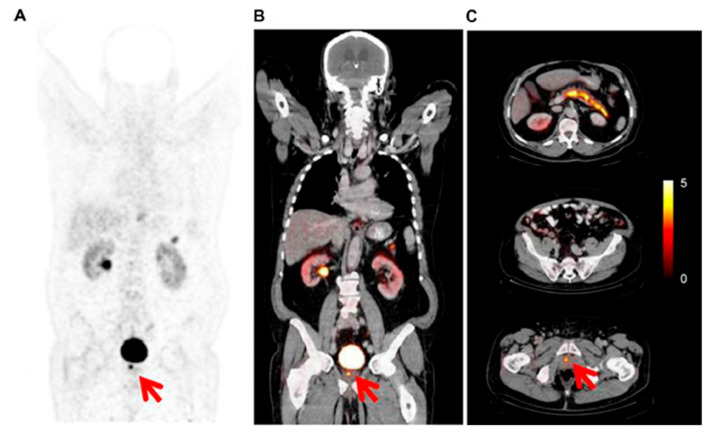
PET/CT GRPRs or bombesin-receptor imaging with ^64^Cu-CB-TE2A-AR06, showing focal uptake of primary prostate cancer (red arrows), below urinary bladder activity, as shown by PET emissive coronal image (**A**), fused corresponding coronal (**B**) and axial slices (**C**) (Copyright © 2021. Wieser, Mansi, Grosu, et al. Positron emission tomography (PET) imaging of prostate cancer with a gastrin releasing peptide receptor antagonist—from mice to men. Theranostics. 2014; 4(4):412–419.65).

GRPR radiopeptides are considered an excellent prospect in the field of theranostics. The encouraging results obtained by the preclinical and the most recent clinical studies suggest that GRPR analogs, and in particular GRPR antagonists, might play in the future an essential role in the diagnosis of prostate cancer at different stages and in the treatment of mCRPC. Figure 3 and Figure 4 show emblematic examples of clinical applications of PET tracers targeting GRPRs in the framework of prostate cancer.

## 4. Theranostic Applications of Radioconjugated Nanomaterials in Prostate Cancer

The significant advancement of nanotechnology has recently led to novel nanomaterials for possible applications in cancer theranostic.

**Figure 5:**
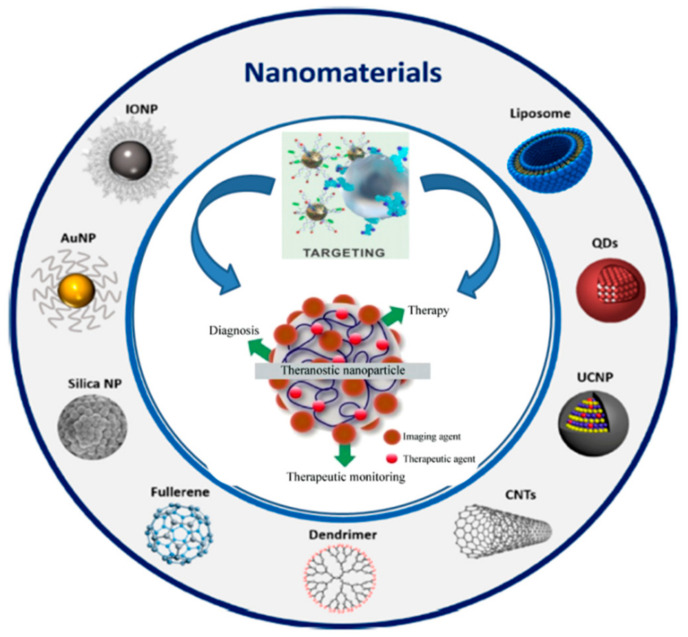
Schematic representation of the potential use of different radioconjugated nanomaterials in theranostics.

The term nanomaterials refer to structures of nanometric size, generally inferior to 100 nm, which may have a biologic origin, as in the case of lipid-based systems, lactic acid or dextran, as well as predominant inorganic characteristics, as in the case of polymers, silica, metals or carbon lattices [40]. One of nanomaterials’ most fascinating properties lies in the possibility of functionalizing their wide surface with specific ligands, thus generating nanosized targeted vehicles of both imaging and therapeutic agents [96]. Figure 5 illustrates the variety of nanomaterials suitable for theranostics.

The theranostic potential resulting from functionalized nanomaterials’ conjugation with specific radionuclides is enormous, going from SPECT and PET imaging to alpha/beta-particle targeted therapy. Concerning prostate cancer, most of the studies exploring the theranostic field of application of radiolabeled nanoparticles have been performed with gamma-emitting radionuclides to follow the in vivo biodistribution and quantify the targeted delivery of nanostructure-based therapies through SPECT imaging.

In recent work, Yari et al. proposed a theranostic approach with promising results in PSMA-expressing prostate cancer based on preformed liposomes functionalized through the addition of a lipopolymer targeting PSMA into the outer surface [95]. The theranostic potential of liposomes, loaded with doxorubicin as a therapeutic agent and labeled with ^99m^Tc for SPECT imaging, was tested in 2 different cell lines having differential PSMA-expression according to both immunoblotting and ^18^F-labeled PSMA ligand uptake [47].

Similar favorable results have been reported with 170 nm-long titanate nanotubes loaded with docetaxel on hormone-refractory prostate cancer cell lines [97]. The radiolabeling with ^111^In through the chelating agent DOTA allowed SPECT imaging to assess biodistribution and tumor retention in a mouse model [48].

The above-mentioned studies underlined the efficacy of radiolabeled nanomaterials as nanovectors of chemotherapy, but the therapeutic potential of nanostructures in prostate cancer is extremely wider, including radiosensitization, gene delivery, and immunotherapy [53]. Moreover, plasmonic nanoparticles, particularly the gold nanoparticles (AuNPs) present the unique property of inducing irreversible thermal cellular death by realizing heat when exposed to light, through a phenomenon called localized surface plasmon resonance [53]. Based on this knowledge, Jiménez-Mancilla et al. elaborated a multifunctional theranostic radiopharmaceutical for prostate cancer centered on ^99m^Tc/^177^Lu-AuNPs functionalized with a bombesin (BN) analog and suitable for both plasmonic photothermal therapy after laser irradiation and targeted radiotherapy, thanks to the beta-particle emission of ^177^Lu and the internal conversion/Auger electron emission of ^99m^Tc [49]. Similarly, Silva et al. chose a BN analog for functionalization and managed to combine targeted AuNPs with DOTA to anchor metal ions, specifically Gd3+ and ^67^Ga, for MRI and SPECT imaging, respectively. The authors reported a favorable radiosensitization profile and concluded that the replacement of ^67^Ga with ^68^Ga, ^90^Y, ^177^Lu, or ^165^Er may offer the advantage of combining SPECT or PET imaging with radionuclide therapy in GRPR-positive prostate cancer [50]. A further promising theranostic application based on AuNPs has been described by Moeendarbari et al. who synthesized injectable brachytherapy nanoseeds by incorporating palladium-103 (^103^Pd) onto hollow AuNPs of about 120 nm. The retention of the nanoseeds after direct injection into a prostate cancer xenograft model was monitored by SPECT/CT thanks to the low energy X-ray emission of ^103^Pd, while the assessment of the therapeutic efficacy was demonstrated through an ^18^F-FDG PET stud [51].

As concerning PET imaging, only very few studies investigated the possibility of conjugating nanomaterials with positron-emitting radionuclides for prostate cancer theranostic purposes.

Chen et al. developed a first-in-kind ultrasmall, PSMA-targeting dual-modality (PET/optical) imaging platform based on previously demonstrated silica nanoparticles [98], with promising theranostic potential in prostate cancer care. The authors covered the ~4 nm amorphous silica core of the nanoparticle with a functional shell comprising a protective polyethylene glycol (PEG) layer to prevent aggregation, a PSMA inhibitor to target prostate cancer cells actively, and a radionuclide chelator, specifically deferoxamine (DFO), to radiolabel with the positron-emitting radionuclide ^89^Zr. Preclinical in vivo PET imaging, ex vivo biodistribution studies and dosimetry revealed favorable pharmacokinetics, favorable renal clearance profiles and low off-target localizations, suggesting that such nanoparticles may represent a versatile theranostic tool in prostate cancer management, ranging from nuclear medicine imaging and image-guided surgery to alpha/beta-particle targeted therapy [52].

The indirect therapeutic potential of nanomaterials has been recently studied in a xenograft mouse model of hypoxic human prostate cancer by Xiang et al. [53], who quantified the oxygenation obtained with nanoscale perfluorocarbon droplets, by using ^18^F-fluoroazomycin-arabinoside (FAZA)-PET imaging, as reported in a previous study [99]. The radioconjugation of such oxygenating nanoemulsions with a beta-particle emitting radionuclide, for instance, ^177^Lu, might further improve the therapeutic effects mediated by reactive production oxygen species (ROS), offering at the same time the possibility of directly imaging the distribution of the nanomaterials through SPECT imaging.

These promising preclinical studies revealed the enormous theranostic potential of radioconjugated nanostructures and highlighted the need for further investigations aimed at acquiring the first clinical experiences in this new emerging research field.

It has to be highlighted, in fact, that despite the fast progress, today no nanoparticle theranostics is so developed to meet clinical standards. Each nanoplatform has its promises and advantages, but meanwhile, has its disadvantages to be overcome. These include the long-term toxicity issues, the cost of gold nanoparticles, their non-biodegradable nature, and some nanoparticles’ oversize. In addition to achieving and validating the nanoscale integration of imaging and therapeutic functions, it is of considerable importance to demonstrate the benefits and synergy of such a combined approach. In theory, an NP-based theranostic agent can deliver therapeutics to a diseased area and can use its imaging function to improve diagnosis and to monitor therapeutic response.

The major obstacle to nanomaterials’ clinical use is the potential long-term safety concerns of these nanomaterials, especially for the non-biodegradable ones that could be eventually retained inside the body for a long time after administration. Although many studies have verified nanomaterial’s short-term safety, their long-term chronic toxic effects remain to be more systematically investigated. Furthermore, it is still unclear how they interact with the immune system. Thus, more systematic studies are still needed to determine the clinical safety of these nanomaterials.

## 5. Future Perspectives and Conclusions

The term “theranostics” defines research efforts to combine diagnostic and therapeutic capabilities into a single agent. The rationale is that close conjunction of diagnosis and therapeutics phases could provide more specific patient-centered therapeutic protocols, thus allowing them to reach improved prognoses. Complex pathologic conditions such as cancer are strongly heterogeneous and existing therapeutic approaches turned out to be really effective only for limited patient subpopulations and at selected stages of the disease.

The main hurdles in cancer treatment still remain the delayed diagnosis and the inability to deliver efficiently therapeutic chemicals to the targeted tumor site without concomitant healthy tissue damage [100]. In the field of nuclear oncology, this application is attracting increasing attention as a targeted, safe, and efficient therapeutic strategy in the era of personalized medicine. Molecular alterations in malignant disease result in the altered expression of various targets that could be used for diagnostic molecular imaging and treatment thanks to specifically selected radiopharmaceuticals.

In recent years, PSMA has emerged as the “star target” for mCRPC theranostics. However, it has to be underlined that prostate cancer cells may present heterogeneous or low expression of PSMA, especially in the advanced stage resulting in poorly responsive to PSMA-directed RLT. It has been proposed that dual tracer PET/CT with ^68^Ga-PSMA-11/^18^F-FDG might be valuable for an accurate characterization of mCRPC patients before systemic therapy [53,101]. Furthermore, already mentioned evidence indicates that PSMA is not exclusively expressed by prostate cancer, leading to unwanted off-targeted effects of PSMA RLT through beta/alpha emitters [85]. In this scenario, it is reasonable to hypothesize that molecular targets other than PSMA, such as GRPRs and PARP-1, might present a crucial role as novel therapeutic options for mCRPC in the next future.

In light of the above, it is gaining more and more consideration from the scientific community a vision of mCRPC as a multi-faceted and “fluid” clinical entity, which needs different approaches at different stages of evolution. Metabolic and molecular PET probes, providing the unique opportunity of getting an insight into the biological complexity of mCRPC, might be used in a singular or combined fashion thus enabling clinicians to move, through multimodality theranostic imaging, towards a personalized therapeutic approach.

## Figures and Tables

**Table 1:** Summary of the main manuscripts on the prognostic and theranostic applications of positron emission tomography (PET) tracers in mCRPC.

Authors	Year	Type of Study	Radiotracer	Target	Comment
Murray et al. [16]	2017	Phase I open-label clinical trial	^18^F-NaF	Newly formed bone	Incorporation of ^18^F-NaF in bone metastases correlated with that of ^223^Ra-dichloride, thus PET/CT with ^18^F-NaF might be utilized for targeted alpha therapy response prediction and dosimetric calculation.
Kairemo et al. [17]	2015	Retrospective, single-center	^18^F-NaF	Newly formed bone	PET/CT with ^18^F-NaF performed at baseline and after the 6th cycle of ^223^Ra-dichloride therapy correlated with PSA changes and resulted useful for monitoring response to targeted alpha therapy.
Khawar et al. [18]	2019	Retrospective, single-center	^68^Ga-DOTA^ZOL^	Osteoclastic bone resorption	PET/CT with ^68^Ga-DOTA^ZOL^ enables the visualization of mCRPC and bronchial carcinoma bone metastases and can be utilized for provisional dosimetry before therapy with ^177^Lu-DOTA^ZOL^
Khawar et al. [19]	2019	Retrospective, single-center	^177^Lu-DOTA^ZOL^	Osteoclastic bone resorption	^177^Lu-DOTA^ZOL^, presenting high absorbed dose in bones and low kidney dose, represents a promising therapeutic agent for skeletal metastases from mCRPC.
Fernandez et al. [20]	2019	Prospective, single-center	^177^Lu-DOTA^ZOL^	Osteoclastic bone resorption	^177^Lu-DOTA^ZOL^ is safe and presents a favorable therapeutic index compared to other radiopharmaceuticals applied for the management of bone metastases.
Kwee et al. [21]	2014	Prospective, single-center	^18^F-choline	Cell membrane biosynthesis	PET-derived volumetric parameters, such as MATV and TLA, correlated with PSA level and represent prognostic factors on overall survival in mCRPC.
Lee et al. [22]	2016	Prospective clinical study	^18^F-choline	Cell membrane biosynthesis	MATV changes on PET/CT, performed at baseline and after three months of therapy, correlated with time to PSA progression in mCRPC subjects submitted to systemic therapy (antiandrogens, sipuleucel-T, chemotherapy, ^223^Ra-dichloride).
Caroli et al. [23]	2018	Prospective clinical study	^18^F-choline	Cell membrane biosynthesis	Overall burden of metabolically active disease (i.e., MATV and TLA calculated on baseline PET/CT) resulted useful to predict mCRPC patients’ outcome after therapy with 2nd generation antiandrogens.
Filippi et al. [24]	2020	Retrospective, single-center	^18^F-choline	Cell membrane biosynthesis	Baseline PSA levels and PET-derived parameters (i.e., TLA and number of lesions) correlated with overall survival of mCRPC patients treated with ^223^Ra-dichloride, TLA resulted independent predictor in multivariate analysis.
Fox et al. [25]	2018	Prospective, single-center	^18^F-FDG	Glycolytic pathway	Patients affected by mCRPC were submitted to dual tracer PET/CT with ^18^F-FDG and ^18^F-FDHT for evaluating androgen receptor (AR) and glycolytic (Gly) status before therapy with 2nd generation antiandrogens. Imaging phenotypes characterized by no-AR expression and positive Gly had the worse prognosis.
			^18^F-FDHT	Androgen receptor	
Bauckneht et al. [26]	2019	Retrospective, single-center	^18^F-FDG	Glycolytic pathway	PET with ^18^F-FDG performed before and after ^223^Ra-dichloride therapy helped identify patients with less favorable prognostic factors (high MTV) and for monitoring response to treatment.
Holland et al. [27]	2010	Pre-clinical	^89^Zr-DFO-J591	PSMA	Immuno-PET with ^89^Zr-DFO-J591 resulted capable of detecting PSMA-expressing tumor xenograft in mice.
Hofman et al. [28]	2018	Single-arm, single-center, phase 2 trial	^68^Ga-PSMA-11	PSMA	Pre-treatment PET with ^68^Ga-PSMA-11 showing at least 1 site of metastatic disease with PSMA uptake was utilized as enrollment criterion for ^177^Lu-PSMA-617 RLT.
Seifert et al. [29]	2020	Retrospective studies	^68^Ga-PSMA-11	PSMA	Quantitative parameters (i.e., PSMA_average_) calculated on PET/CT with ^68^Ga-PSMA-11 resulted useful for selecting mCRPC patients before RLT with ^177^Lu-PSMA-617.
Bouvet et al. [30]	2016	Pre-clinical	^18^F-DCFPyL	PSMA	The radiocompound, obtained via direct radiofluorination, was capable of binding with high specificity PSMA-expressing tumor xenografts in nude mice, with a tumor-to-blood pool ratio of 8.3 at 60 min.
Müller et al. [31]	2019	Pre-clinical	^152^Tb-PSMA-617	PSMA	PSMA-617, labeled with the positron-emitter ^152^Tb, was successfully used to image PSMA-positive tumor xenografts in mice and to visualize metastases in a mCRPC patient.
Müller et al. [32]	2019	Pre-clinical	^161^Tb-PSMA-617	PSMA	PSMA-617, labeled with ^161^Tb, emitting both photons and Auger electrons, was tested as theranostic agent in vitro and in mice bearing PSMA-positive xenografts, showing superior results as compared to those obtained with ^177^Lu-PSMA.
Dehdashti et al. [33]	2005	Clinical trial	^18^F-FDHT	AR	PET/CT with ^18^F-FDHT resulted in identifying AR status in patients with advanced prostate cancer; tracer binding was selectively blocked by the administration of flutamide.
Scher et al. [34]	2010	Phase I-II study	^18^F-FDHT	AR	PET/CT with ^18^F-FDHT was applied for determining the safety and antitumor activity of enzalutamide.
Vargas et al. [35]	2018	Prospective, multi-center study	^18^F-FDHT	AR	PET/CT with ^18^F-FDHT was proved to be highly repeatable with high inter-observer reproducibility, thus presenting potential usefulness for AR status monitoring during hormonal treatments.
Zhou et al. [36]	2018	pre-clinical	[^18^F]WC-DZ-F	PARP-1	The synthesized PARP-1 radioligand resulted in being a suitable PET imaging agent for assessing PARP-1 expression in prostate cancer with high uptake in PC-3 cells and favorable biodistribution in xenograft tumor mice
Zhang-Yin et al. [37]	2020	Pre-clinical	^68^Ga-AMBA	GRPR	^68^Ga-AMBA showed good tumor uptake, high tumor-to-background contrast using PC3 cell line.
Dam et al. [38]	2015	Pre-clinical	^55^Co-NOTA-AMBA	GRPR	^55^Co-NOTA-AMBA in PC3 xenografted mice was found to be superior, for PET/CT imaging, compared to ^68^Ga-NOTA-AMBA, since it showed a better tumor-to-organ ratio
			^68^Ga-NOTA-AMBA		
Maddalena et al. [39]	2009	Pre-clinical	^177^Lu-AMBA	GRPR	^177^Lu-AMBA showed good radiotherapeutic efficacy in LNCaP, DU145, or PC-3 tumor–bearing male nude mice and was capable of identifying tumors in vivo.
Zhang et al. [40]	2007	Pre-clinical	^67^Ga-DOTAPESIN	GRPR	^67^Ga/^177^Lu-DOTAPESIN showed high uptake in human prostate tumor xenografts and in murine GRPR-positive organs, PET images demonstrated that ^68^Ga-DOTA-PESIN accumulates predominantly in PC-3 tumor, pancreas, and kidneys.
			^68^Ga-DOTAPESIN		
			^177^Lu-DOTAPESIN		
Wieser et al. [41]	2014	Clinical	^64^Cu-CB-TE2A-AR06	GRPR	In 3 out of 4 patients with newly diagnosed prostate cancer, ^64^Cu-CB-TE2A-AR06 was able to visualize tumors with high-contrast.
Maina et al. [42]	2015	Pre-clinicalClinical	^67^Ga-SB3	GRPR	^68^Ga-SB3 showed, in patients affected by disseminated prostate and breast cancer submitted to PET/CT, pathological uptake in, respectively, 55% and 50% of patients. ^67^Ga-SB3 showed good pharmacokinetics in mice.
			^68^Ga-SB3		
Lymperis et al. [43]	2018	Pre-clinical	^111^In-SB3	GRPR	The study aimed to explore the theranostic potential of ^111^In-SB3 for SPECT imaging and ^177^Lu-SB3 for radionuclide therapy in GRPR-positive PC-3 xenografts.
			^177^Lu-SB3		
Chatalic et al. [44]	2016	Pre-clinical	^68^Ga-JMV4168	GRPR	In PC-3 tumor-bearing mice, the co-injection of a neutral endopeptidase inhibitor led to enhanced PC-3 tumor signal intensity in PET imaging with ^68^Ga-JMV4168, as well as regression of tumor size and increased survival rate following radionuclide therapy with ^177^Lu-JMV4168.
			^177^Lu-JMV4168		
Dalm et al. [45]	2017	Pre-clinical	^68^Ga-NeoBOMB1	GRPR	In a mouse model, ^68^Ga-NeoBOMB1 for PET/CT imaging and ^177^Lu-NeoBOMB1 for radionuclide therapy reported, respectively, good visualization of the tumor tissue in PET images and high uptake in PC-3 cells.
			^177^Lu-NeoBOMB1		
Khurt et al. [46]	2019	Clinical	^68^Ga-RM2	GRPR	Thirty-five patients with mCRPC underwent PET/CT with ^68^Ga-RM2. Among these, 4 underwent therapy with ^177^Lu-RM2, showing high tumor uptake and rapid clearance from normal organs and suggesting its suitability for radionuclide therapy in patients with mCRPC.
			^177^Lu-RM2		
Yari et al. [47]	2019	Pre-clinical	P^3^-liposomes labeled with ^99m^Tc and loaded with doxorubicin	PSMA	Liposomes carrying the lipopolymer P^3^ can be used for targeted delivery of therapeutics/diagnostics to advanced/metastatic PSMA^+^ prostate cancer tumors.
Loiseau et al. [48]	2017	Pre-clinical	TiONts-DTX-DOTA [^111^In]	22Rv1	After intratumoral injection, more than 70% of TiONts nanovectors were retained within the tumor for at least 7d with a significant reduction of tumor growth compared with free DTX
Jiménez-Mancilla et al. [49]	2013	Pre-clinical	^99m^Tc/^177^Lu-AuNP-Tat (49–57)-Lys^3^-BN	GRPR	The nanosystem showed properties suitable for both plasmonic photothermal therapy and targeted radiotherapy with β-particle, IC electrons and Auger electrons
Silva et al. [50]	2019	Pre-clinical	^67^Ga-AuNP-Gd-BBN	GRPR	In addition to the favorable radiosensitization profile exhibited by bimodal MRI/SPECT AuNPs, the replacement of ^67^Ga with ^68^Ga, ^90^Y, ^177^Lu or ^165^Er could offer the possibility of combining SPECT, PET or MR imaging with β-particle targeted therapy
Moeendarbari et al. [51]	2016	Pre-clinical	^103^Pd@Au-nanoseeds	Not applicable	Au-nanoseed-based brachytherapy has the potential to provide a theranostic solution for unresectable tumors exploiting the γ-emission of ^103^Pd for SPECT imaging
Chen at al. [52]	2020	Pre-clinical	^89^Zr-DFO-PSMAi-PEG-Cy5-C’ dots	PSMA	PSMA-targeting C’ dots could represent a highly versatile theranostic tool for prostate cancer management, from imaging and image-guided surgery to treatment planning and α/β-particle targeted therapy
Xiang et al. [53]	2019	Pre-clinical	^18^F-FAZA	Hypoxic regions of tumors	PET imaging with the hypoxia radiotracer ^18^F-FAZA can be used to assess reoxygenation of hypoxic tumors obtained with perfluorocarbon nanodroplets

Abbreviations: MATV—metabolically active tumor volume; TLA—total lesion activity; ^18^F-NaF—sodium fluoride; mCRPC—metastatic castration-resistant prostate cancer; ^18^F-FDHT—18fluorodihydrotestosterone; ^18^FDG—18fluorodeoxyglucose; min—minutes; h—hours; PSMA—prostate specific membrane antigen; RLT—radioligand therapy; PARP-1—poly (ADP-ribose) polymerase-1; PET—positron emission tomography; PC-3—prostate cancer 3; WC-DZ-F—olaparib analogue; AMBA—Do3A-Ch2Co-G-(4-aminobenzoyl)-QWAVGHLM-NH2; GRPR—gastrin-releasing peptide receptor; ^64^Cu-CB-TE2A-AR06—(^64^Cu-4,11-bis(carboxymethyl)-1,4,8,11-tetraazabicyclo(6.6.2)hexadecane)-PEG_4_-D-Phe-Gln-Trp-Ala-Val-Gly-His-Sta-LeuNH_2_]; SB3—(DOTA-paminomethylaniline-diglycolic acid-DPhe-Gln-Trp-Ala-ValGly-His-Leu-NHEt); JMV4168—DOTA-βAla-βAla-[H-D-Phe-Gln-Trp-Ala-Val-Gly-His-Sta-Leu-NH2; RM2—DOTA coupled to D-Phe-Gln-Trp-Ala-Val-Gly-His-Sta-Leu-Nh2 with a cationic spacer 4-amino-1-carboxymethyl-piperidine; P^3^—lipopolymer comprising of PSMA ligand; polyethylene glycol (PEG2000) and palmitate; TiONts—titanate nanotubes; DTX—docetaxel; DOTA-1,4,7,10-tetraazacyclododecane-1,4,7,10-tetraacetic acid; d-days; AuNP—gold nanoparticle; Lys—lysine; BN/BBN—bombesin; IC—internal conversion; Gd—gadolinium; MR—magnetic resonance; SPECT—single photon emission computed tomography; DFO—deferoxamine; Cy—cysteine; FAZA—fluoroazomycin-arabinoside; 22Rv1—hormone-refractory prostate cancer cell line. Half-lives of the radionuclides cited in the Table: ^18^F = 110 min; ^89^Zr = 78.41 h; ^68^Ga = 68 min; ^55^Co = 17 h; ^177^Lu = 6.7 days; ^64^Cu = 12.70 h; 111In = 67 h; ^152^Tb = 17.5 h; ^161^Tb = 6.89 days.
